# The Physical Side of Social Regeneration

**Published:** 1917-11-17

**Authors:** 


### THE BURKE RELIEF FOUNDATION, NEW YORK.

### The Physical Side of Social Regeneration.

A typical example of American progress and the American's serious regard for the welfare of the citizen is to be seen in the convalescent home at White Plains, some five-and-twenty miles from New York. Founded and endowed by the munificence of one man, the late John Masterson Burke, of that city, it aims not only to restore to health those recovering from surgical operation and illness, but to prevent sickness and invalidism by a short period of rest, appropriate food, and freedom from worry and care in pleasant and healthful surroundings.

Opened in April 1915, it is ideally situated in a rural district in the midst of a well-wooded estate of sixty acres. The buildings are in colonial style, comprising eleven blocks enclosing a large central quadrangle, and ample provision has been made for future extensions. In addition, there are two separate establishments for boys and girls between the ages of ten and sixteen and a home for coloured women convalescents.

### What the Foundation Accomplishes.

The accommodation at White Plains is for 120 men and 100 women, whilst, with the branch institutions, the total number of beds available amounts to 270. During the first year about 5,000 patients passed through the institution, thus approximately doubling the convalescent provision for New York City. But the aims of the executive go deeper than merely providing a short period of rest after illness in a city hospital. "Our principal aim," they state in a descriptive booklet, "is to increase the proportion of constructive and also of preventive convalescence. These terms, while not very definite, apply valuably in practice. Giving a few weeks of pleasant residence to a woman having fair connections and support and no planned productivity may be thought of as passive convalescence. Contrast this with the restoration and replacement thus in work and contentment of the family wage-earner or depleted mother nearing permanent breakdown. Cardiacs having records of months of each year in hospitals and who, by directed rest plus occupation-therapy are upbuilt for long periods of continuance in reasonable *productiveness* furnish good examples of the more difficult but worthy forms of this preventive and constructive effort. A complete cycle of co-operations may be thus outlined: A fatigued and failing worker sent to the country for recuperation, returns for a year or two to fair productiveness, fails again, and applies for re-admission, is urged to have a more thorough diagnosis; serious underlying complications are found, operation, followed by full restoration in the convalescent institution, with home and suitable work arranged for the return."

### Cardiac Lesions a Special Feature.

The care of those suffering from cardiac lesions is a special feature of the work of the foundation. In the first year of the institution 273 were cared for, and many others were refused admission. The men are trained to do carefully graded work in cardiac classes; their stay is a fairly long one (on an average five weeks), and after passing a rigid physical examination they are sent to work in the city shops. A workshop is also run in connection with the "Trade School for Cardiac Convalescents," where the men are taught trades such as are adapted to their physical condition. In the boys' branch the opportunities for success are naturally greater. Young heart muscle compensates more readily than does old, and by carefully graduated exercises it has been found possible in almost all cases to restore the young cardiac to two-thirds normal activity.

### Thoughtfully Chosen and Directed Occupations.

Occupation has over here, in the case of our disabled and shell-shock patients, become to be recognised as a most important factor in promoting a speedy recovery, and at White Plains a regular system of work cure is in vogue. Naturally, with an average stay of only three weeks there are certain difficulties to be overcome. "Occupation, thoughtfully chosen and directed, is becoming our most effective instrument in many classes—notably in heart disease, hyperthyroidism, the many and various nerve and mental borderline conditions, drug addictions, the temperamentally restless and unstable—and those in general needing extended residence, as with certain slow-healing wounds, cripplings, graver malnutritions, etc. And especially are we finding prescribed occupation to be the main arm in lifting the disheartened, the defibred, the overtreated and pampered back into confidence and the desire to rejoin the producers in life." The work is very varied, and suitable to all grades of physical and mental development. There are classes in basketry, sewing and fancy work, looms, carpentry, cobbling, tailoring, etc.; others are employed in tending the extensive grounds, or give assistance in the office and library, or devote themselves to the care and discipline of their fellow inmates. After the convalescent period has come to an end an effort is made, with the assistance of various New York charitable societies, to secure suitable employment, and a follow-up system supervises where necessary the home recovery of the patient.

### Some Chronic Cases and Efficiency.

Though the rules of the institution do not permit of the treatment of chronic cases, yet many who are more or less permanently unfit find a temporary home at White Plains. If there seems any chance of restoring them to a reasonable economic efficiency they are gladly received. "Keeping subnormal people at reasonable limited production seems to us a most worthy function." That, as the report shows, many who had through long invalidism ceased to think in terms of work should, after a stay of seven weeks or so, be eager and restless to leave and "get a job" is the highest testimony that can be given to the efficacy of this system of social regeneration.

